# Perspectives on Osteoarthritis Treatment with Mesenchymal Stem Cells and Radix Achyranthis Bidentatae

**DOI:** 10.14336/AD.2023.0817

**Published:** 2024-05-07

**Authors:** Xingyan An, Jiao Wang, Ke Xu, Robert Chunhua Zhao, Jiacan Su

**Affiliations:** ^1^Institute of Translational Medicine, Shanghai University, Shanghai, China.; ^2^Organoid Research Center, Shanghai University, Shanghai, China.; ^3^Institute of Basic Medical Sciences Chinese Academy of Medical Sciences, School of Basic Medicine Peking Union Medical College, Beijing, China.; ^4^Center for Excellence in Tissue Engineering, Chinese Academy of Medical Sciences, Beijing Key Laboratory of New Drug Development and Clinical Trial of Stem Cell Therapy, Beijing, China.; ^5^School of Life Sciences, Shanghai University, Shanghai, China.; ^6^Department of Orthopaedics, Xinhua Hospital, Shanghai JiaoTong University School of Medicine, Shanghai, China.

**Keywords:** mesenchymal stem cells, Radix Achyranthis Bidentatae, osteoarthritis, estrogen, intestinal flora

## Abstract

Knee osteoarthritis, a widespread degenerative condition, impacts a younger population and leads to high disability rates. Nature often provides solutions for aging and disease prevention. Mesenchymal stem cells (MSCs) and Radix Achyranthis Bidentatae (AB) are natural substances with potential. MSCs can transform into various tissues, alleviating symptoms by releasing factors and miRNA, potentially slowing osteoarthritis progression. AB's compositions target knee joint cells, enhancing internal conditions and joint function. Both MSCs and AB share mechanisms for immune regulation, reducing cartilage apoptosis, promoting chondrocyte formation, and addressing osteoporosis. They also influence estrogen and gut flora. This article reviews their roles in treating osteoarthritis, discussing apoptosis reduction, chondrocyte growth, bone enhancement, angiogenesis, and regulation of estrogen and intestinal flora. It explores their relationship and suggests AB's potential in stimulating mesenchymal stem cell repair for knee osteoarthritis treatment.

## 1. Introduction

Osteoarthritis (OA) has become a prevalent disease in the modern world, significantly affecting the quality of life for individuals aged 60 and above [1]. Various factors contribute to its development, including biomechanical changes in the knee due to trauma, aging, overweight, wind-cold-dampness intrusion, and estrogen decline in menopausal women [2]. In those suffering from osteoarthritis, progressive damage to the articular cartilage leads to severe pain, limited mobility, and potential disability [3]. The advent of Mesenchymal Stem Cells (MSCs), a novel biological drug, has gained global attention in recent years. MSCs have emerged as a breakthrough in treating degenerative and immune-related diseases, including osteoarthritis [4-6]. Their primary pharmacological mechanisms encompass promoting cell regeneration, reducing cell apoptosis, modulating the immune response, and improving the microenvironment through the release of exosomes. Radix Achyranthis Bidentatae (AB), known as "Niu Xi" in Chinese due to its resemblance to an ox's knee joint, holds a significant place in traditional Chinese medicine for treating knee ailments. AB's traditional use aligns with the principle that substances resembling specific body parts can aid in their healing [7]. The Chinese pharmacological book "Ben Cao Gang Mu" attributes AB with the ability to unblock vascular congestion, nourish the liver and kidneys, and enhance the quality of tendons and bones. Since the liver relates to bones and the kidney with meridians in traditional Chinese medicine, AB's potential in treating osteoarthritis is evident [8-9]. Both MSCs and AB show efficacy in addressing the damage to the triple-energizer mesenchymal stem cell system caused by osteoarthritis [10]. Consequently, the treatment of osteoarthritis garners significant attention. It's important to recognize that knee osteoarthritis isn't solely a cartilage-related ailment; it encompasses various components of the knee, including subchondral bone, articular cartilage, joint capsule, fat pad, synovium, fascia, synovial fluid, peripheral nerves, blood vessels, lymph, muscles, and tendons. Additionally, it is intertwined with the body's endocrine system, immune response, and intestinal flora [11].

As the body ages, hormonal levels decrease, and the elasticity of fascial tissue weakens. Particularly, if the triple-energizer mesenchymal stem cell system around the knee joint is compromised, issues may arise. For instance, when tissues like the joint capsule, fat pad, fascia, and tendons deteriorate, cartilage's nutritional channels become restricted, leading to degradation, apoptosis, and depolarization. This situation demands the reconstruction of a collateral circulation supply system, triggering an increase in angiopoietin levels to signal damaged cartilage and promote nutrient supply and metabolism. Unfortunately, this can inadvertently breach the boundary between articular cartilage and bone, introducing nutrients and osteoclasts simultaneously. The immune system's involvement can worsen inflammation and induce cartilage apoptosis. The transformation of osteoclasts into osteoclast-like functional cells with immune escape further exacerbates cartilage ossification, ultimately causing knee joint damage.

The description provided highlights how the pathological changes in knee joint cartilage represent a complex self-healing process, which can also pose challenges [12]. Consequently, it's crucial to prioritize the restoration of the triple-energizer mesenchymal stem cell system around the knee joint before specific blood vessels approach the joint cavity. Methods like local warmth maintenance, targeted muscle strength training, and the restoration of fascia elasticity can all effectively slow down the progression of arthritis.

Moreover, both MSCs and AB offer effectiveness in arthritis treatment. Stem cells are derived primarily from sources such as body fat, placenta, umbilical cord, and bone marrow. These cells possess remarkable potential for differentiating into various tissues [13]. Additionally, stem cells demonstrate regenerative and reparative capabilities, immune modulation, microcirculation enhancement, homeostasis maintenance, and chondrocyte protection. AB, which is derived from the Achyranthes plant of the Amaranthaceae family, has gained prominence due to its ability to treat "bone pain" by nourishing the kidneys and promoting blood circulation. Gradually, it has become a widely used traditional Chinese medicine for osteoarthritis treatment. Its pharmaceutical value lies in strengthening tendons and bones, nourishing the liver and kidneys, enhancing blood circulation, and facilitating downward blood movement. AB contains various chemical compounds, including polysaccharides, saponins, ecdysterone, flavonoids, peptides, organic acids, and various trace elements. These compounds collectively contribute to its efficacy in treating osteoarthritis [14].

## 2. Reduce cartilage apoptosis through immunomodulation

In the process of treating osteoarthritis, it is of equal significance to address inflammation and safeguard cartilage. During the initial stages of osteoarthritis development, the immune response operates in a compensatory manner, striving to eliminate irregular factors within the knee joint. In this phase, inflammation can be reversed when these abnormal factors are effectively removed. However, as the immune response persists over time, the internal knee joint environment remains in an imbalanced state. This instability can disrupt the original ecological balance, potentially leading to an eruption of local immune factors and triggering a cascade of issues including autophagy dysregulation and chondrocyte apoptosis. Consequently, the progression of osteoarthritis ensues.

To combat this progression, the introduction of exogenous drugs becomes essential. These drugs work to reduce the concentration of immune factors, dampen the immune response, and counteract the apoptosis and degradation of cartilage. This multi-faceted approach targets inflammation and cartilage protection to effectively manage and mitigate the course of osteoarthritis.

### 2.1 Immunomodulation of MSCs

MSCs play a crucial role in achieving immunomodulation during osteoarthritis treatment. When used for treating osteoarthritis, MSCs could suppress inflammation and protect articular cartilage. Research indicates that upon introduction of MSCs to osteoarthritis treatment, there is a significant reduction in the levels of several key biomolecules, including MMP-3, IL-6, TIMP-1, and TNF-α. Furthermore, the levels of inflammation-related cytokines like IFN-γ, IL-2, IL-8, IL-10, and IL-12 are simultaneously affected. This showcases MSCs' ability to modulate the inflammatory response [15-16].

One notable component of MSCs' effectiveness is their exosomes, which are small vesicles containing various bioactive molecules. These exosomes can enhance chondrocyte protection by inhibiting the NF-κB pathway [15-16]. A specific exosomal miRNA, miR-361-5p, demonstrates its efficacy in alleviating osteoarthritis by targeting DDX20 and deactivating the NF-κB signaling pathway. In chondrocytes affected by IL-1β, DDX20 expression increases while miR-361-5p expression decreases. In this scenario, miR-361-5p directly targets DDX20, reducing its expression and subsequently suppressing matrix metalloproteinase (MMP) levels. This process mitigates IL-1β-induced inflammation, curbing chondrocyte damage and inhibiting the NF-κB pathway. Ultimately, this exosome-mediated regulation counters the adverse effects of DDX20 overexpression on IL-1β-induced chondrocyte damage [17].

Additionally, another miRNA found in MSC exosomes, miRNA-210, contributes to the suppression of pro-inflammatory cytokines, the regulation of mitochondrial autophagy via drp1, and the inhibition of chondrocyte apoptosis and MMP expression. In cases involving lipopolysaccharide (LPS)-induced chondrocyte damage, miRNA-210 hampers the expression of tumor necrosis factor receptor superfamily member 21 (Tnfrsf21), thereby weakening the NF-κB pathway. This miRNA promotes chondrocyte proliferation and dampens LPS-induced cell apoptosis [18].

Furthermore, exosomes from MSCs also influence osteoarthritis development by regulating drp1-mediated mitochondrial autophagy and inhibiting chondrocyte apoptosis and MMP expression. In specific studies using advanced glycosylation end products (AGEs) to interfere with rat primary chondrocytes, overexpression of Drp1 reduces the expression of LC3-II/LC3-I and Beclin-1, increases apoptosis in AGEs-interfered chondrocytes, and elevates the expression of MMP-3 and MMP-13. MSC-derived exosomes counteract the AGEs-induced apoptosis increase and prevent the upregulation of MMP-3, MMP-13, and Drp1 in chondrocytes [20].

The role of MSCs and their exosomes in suppressing inflammation, protecting chondrocytes, and influencing various pathways holds significant promise for osteoarthritis treatment.

**Table 1:** Convergent Effects: Stem Cells and AB in Arthritis - Inflammation Inhibition, Matrix Regulation, and Chondrocyte Apoptosis Reduction, with Parallel Immune Modulation.

Immunomodulation and reduction of cartilage degeneration	
**MSCs**	AB
**Inhibit IL-2**	Inhibit IL-2
**Inhibit IL-6**	Inhibit IL-6
**Inhibit TNF-α**	Inhibit TNF-α
**Inhibit IL-12, IL-8, IL-10**	Reverse the imbalance of Th17/Treg
**Downregulate MMP-3**	Downregulate the MMP expression including cyclooxygenase-2, MMP 3, and MMP 9
**Downregulate IFN-γ**	Inhibit the genetic expression of apoptotic genes bax and Bad
**Downregulate TIMP-1**	Suppress p53 and p65 phosphorylation, and phosphorylation and degradation of IκBα
**miR-361-5p can target on DDX20 to ease osteoarthritis damage, and inactivate NF-κB signaling pathway**	Promote the expression of anti-apoptotic protein Bcl-xLand
**Downregulate Tnfrsf21**	Glycolytic upstream Hif-1 and AKT sequential signaling weakens
**Inhibit osteoarthritis induced by IL-1β through miR-214-5p/PPARGC1B axis**	Pkm2, Eno2, Eno2, Pdk1, Pgk2 and Hk2 reduce
**Regulate drp1-mediated mitochondrial autophagy**	Inhibit the expression of the genes Gadd45a, Gadd45g and Mdm2 (the typical target genes of the cell-cycle negative regulatory factors Plk2 and p53)
**SNHG7 gains miR-214-5p as a competitive endogenous RNA through sponge absorption, targeting on PPARGC1B**	Inhibit the mRNA level of Casp1 and Casp12, the components of the apoptotic pathway

### 2.2 Immunomodulation of AB

Research has shown that AB (Radix Achyranthis Bidentatae) has the capacity to effectively reduce the levels of inflammatory cytokines, specifically TNF-α, IL-2, and IL-6, within synovial tissues. This is accomplished by regulating the balance between Th17 and Treg cells, leading to the inhibition of inflammatory factor secretion. AB's action helps counteract the Th17/Treg imbalance, which in turn retards chondrocyte apoptosis in the knee joint. AB influences the expression of collagen and metalloproteinase in knee joint chondrocytes, contributing to its protective effect [21].

The NF-κB transcription factor, comprising Rel A (p65), Rel B, c-Rel, NF-κB1 (p50 and its precursor p105), and NF-κB2 (p52 and its precursor p100), has been identified as a key factor in osteoarthritis pathogenesis. This transcription factor family forms different heterodimers or homodimers, each capable of activating distinct genes. Normally, these dimers exist in an inactive state within the cytoplasm, bound to IKB family proteins to create trimer-inert complexes. When exposed to external stimuli, these NF-κB complexes dissociate. The dimers then migrate to the nucleus, binding to target genes and triggering their function. This process triggers synovial inflammation and indirectly influences downstream regulators of chondrocyte terminal differentiation, thereby affecting cartilage matrix remodeling, chondrocyte apoptosis, and cartilage damage. AB plays a role in suppressing this signaling pathway by influencing genetic expression and protein phosphorylation [22].

Furthermore, AB can inhibit glycolysis and cell apoptosis to protect chondrocyte function through the MAPK/AKT signaling pathway. The MAPK signaling pathway is pivotal for transmitting signals from the cell membrane to the nucleus. This pathway involves a three-tiered enzymatic cascade reaction that regulates various cellular functions. In osteoarthritis, metabolic abnormalities are common. Joint swelling can elevate glucose uptake, intensifying glycolysis and causing cartilage damage. AB intervenes by weakening the glycolytic pathway and suppressing the downstream effects of glycolysis. It also impacts key genes, such as those associated with cell-cycle regulation and apoptosis. By activating the MAPK pathway and inhibiting the AKT pathway, AB reduces glycolysis and creates a more favorable environment for chondrocytes [23] Table 1.

**Figure 1.**
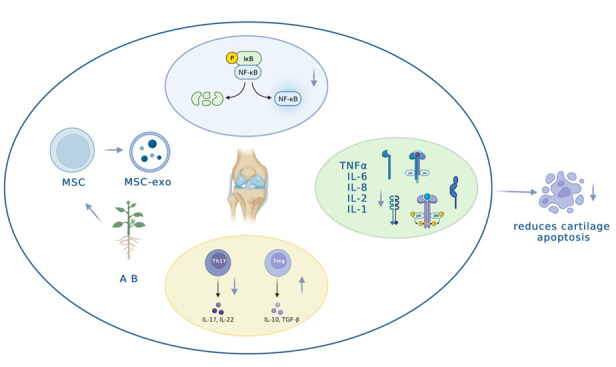
**MSCs and AB reduce cartilage apoptosis through immunomodulation**. In the treatment of knee osteoarthritis, exogenous stem cells have shown the effect of immunomodulation. Stem cells can secrete exosomes with multiple miRNAs that can reverse the imbalance of Th17/Treg, inhibit the secretion of inflammatory factors, weaken the NF-κB pathway, downregulate apoptotic genes, alleviate inflammation, and reduce chondrocyte apoptosis. AB also has immunomodulatory and anti-inflammatory effects in treating knee osteoarthritis. As a plant-derived drug, AB can potentially promote the secretion of exosomes that inhibit inflammation-related factors by acting on in situ stem cells.

In conclusion, both MSCs and AB exhibit the ability to inhibit inflammatory factors, attenuate the NF-κB pathway, and downregulate apoptotic genes. Furthermore, AB has the potential to achieve these effects by influencing in situ stem cells. This multi-faceted approach is illustrated in Figure 1.

## 3. Promote chondrocyte proliferation

Cartilage serves as an essential supportive tissue within the knee joint. Its matrix possesses a gelatinous and robust composition. During knee joint movement, this cartilage matrix acts as a buffer, mitigating pressure and minimizing friction. However, in the context of pathological changes associated with knee osteoarthritis, there is an increase in chondrocyte apoptosis coupled with a decrease in proliferation. This dynamic eventually results in the gradual thinning or complete loss of the cartilage layer, translating to a loss of knee joint functionality. Unfortunately, cartilage has limited regenerative capabilities, making it difficult to naturally regenerate or repair. As a consequence, the promotion of chondrocyte proliferation becomes crucial as it can effectively delay the progression of osteoarthritis and alleviate associated symptoms. In the realm of osteoarthritis treatment, the enhancement of chondrocyte proliferation and the reversal of cartilage thinning have emerged as prominent and essential areas of research focus.

### 3.1 MSCs promote chondrocyte proliferation

In clinical practice, the regeneration of damaged cartilage presents significant challenges, as the inherent regenerative capacity of cartilage is limited. Additionally, obtaining a sufficient quantity of functional chondrocytes is often a difficult endeavor. However, MSCs offer a readily available and abundant resource. As a result, MSCs have gained clinical prominence for their capacity to serve as "chondrocyte seeds" in the process of cartilage regeneration.

MSCs possess the remarkable ability to differentiate into chondrocytes, the specialized cells responsible for forming cartilage. Through a paracrine mechanism, MSCs can also exert beneficial effects on the function of existing articular chondrocytes (ACs). This paracrine communication involves the secretion of signaling molecules and factors that influence the behavior and function of surrounding cells. By participating in this intricate communication network, MSCs contribute to the rejuvenation and restoration of articular cartilage function [24].

#### 3.1.1 Differentiation of MSCs into chondrocytes

MSCs can be influenced by TGF-β3 through the Wnt5a/b-catenin pathway to facilitate cartilage growth. Additionally, MSCs have the capability to activate the Wnt signaling pathway by upregulating the expression of PLCE1, CaMKII-β, and downstream NFATc1, resulting in a heightened expression level of cartilage markers, such as SOX9. This process triggers the activation of IKK/NF-κB signaling pathways, PI3K-PDK-AKT, and atypical Wnt, ultimately leading to cartilage regeneration [25]. Furthermore, MSCs possess the ability to enhance chondrogenic regeneration through the modulation of the TGF-β/Smad2 signaling pathway, achieved via the endothelial-cell proliferation marker CD105. This mechanism supports the formation of cartilage by augmenting the Smad2 signaling pathway [26]. Additionally, miR-892b and the zinc finger transcription factor KLF10, a member of the Krüppel-like transcription factor family, can promote chondrogenesis with MSCs [27].

The process of mesenchymal cell coagulation stands as a pivotal transitional stage before chondrogenic regeneration. Collagen microencapsulation can lead to the upregulation of chondrogenic transcription factors. The immediate elevation of coagulation markers, including peanut lectin, fibronectin, integrin α5, and αv, facilitates the nuclear localization of SOX9 and its binding to COL2A1. This promotes the differentiation of MSCs into cartilage. The substantial expression of Linc-ROR significantly drives chondrogenesis from MSCs in vitro and activates cartilage formation in vivo. MSCs are induced to differentiate into chondrocytes, thereby upregulating the expression of chondrogenic marker genes including col10, sox9, col2, aggrecan, and pthrp. This induction is achieved using microorganisms such as polyhydroxyalkanoate (PHA) and poly(3-hydroxybutyrate-co-3-hydroxyhexanoate) (PHBHHx).

#### 3.1.2 MSC-exosomes can promote chondrocyte proliferation

In co-cultures of chondrocytes and MSCs, cells exhibiting the morphology of chondrocytes tend to display heightened proliferation rates. Stem cells play a role in initiating or sustaining chondrogenesis through the secretion of chondrogenic factors encapsulated in exosomes. These factors could stimulate chondrocyte proliferation, enhance the production of extracellular matrix components (such as type II collagen and cartilage glycosaminoglycan, GAG), and improve the expression of pivotal chondrocyte genes like type II collagen, aggrecan, and SOX-9.

Studies have indicated that specific miRNAs present in MSC-derived exosomes can facilitate various chondrocyte processes, including division, migration, anabolic activity, and anti-inflammatory responses. For instance, the expression of miR-205-5p, miR-381-3p, miR-29a, and miR-29b within MSC exosomes can promote chondrocyte proliferation. In addition, also contribute to chondrocyte proliferation through the miR-205-5p/PTEN/AKT pathway [28]. MiR-381-3p directly inhibits TAOK1 by targeting its 3' untranslated region, consequently suppressing the hippopotamus signaling pathway and fostering cartilage formation [29]. MiR-29a, along with miR-29b, directly targets a segment of the 3' untranslated region of the col2a1 gene, which encodes type II collagen, thereby promoting chondrogenesis [30].

### 3.2 The role of AB in promoting chondrocyte proliferation

Previous studies have demonstrated that AB possesses the ability to enhance the expression of cyclin-dependent kinases (CDKs) and cyclins, thereby regulating the cell cycle and promoting chondrocyte proliferation. Additionally, AB is also known to enhance the secretion of the extracellular matrix, further supporting chondrocyte proliferation.

AB is shown to upregulate the expression of key elements like cyclin D1, β-catenin, Frizzled-2, and Wnt-4, while downregulating GSK-3β (glycogen synthase kinase 3β). This leads to the translocation of β-catenin into the nucleus, enhancing the expression of proteins and mRNAs like cyclin D1, CDK4, and CDK6, and facilitating the transition of chondrocytes from the G1 phase to the S phase. AB activates the Wnt/β-catenin signaling pathway, inducing the G1/S phase transition, and supporting chondrocyte proliferation. In the absence of Wnt signaling, GSK-3β phosphorylation leads to β-catenin degradation and reduced cyclin D1 expression. However, AB promotes the nuclear translocation of non-phosphorylated β-catenin, boosting cyclin D1 expression by activating the Wnt/β-catenin pathway [31].

Furthermore, AB contributes to improved joint adhesion by enhancing the expression of type II collagen, the primary secretory molecule of the extracellular matrix. This helps restore cartilage matrix components, stabilize the structure of the tidal line, and repair cartilage damage. AB extract has the potential to enhance chondrocyte proliferation and activity in knee osteoarthritis treatment. It also influences the cell cycle by decreasing cell growth arrest in the G0/G1 phase and increasing DNA synthesis in the S phase. Additionally, AB extract exhibits the ability to decrease the rate of chondrocyte apoptosis, as demonstrated in apoptosis detection assays. This suggests that AB extract, through its impact on the cell cycle, can potentially promote knee osteoarthritis chondrocyte proliferation, repair damaged cartilage, and alleviate associated symptoms. In certain experiments, human knee osteoarthritis chondrocytes are cultured, and drug-containing serum from human subjects is utilized for testing. This experimental design offers a realistic depiction of AB extract's metabolism within the human body, providing a robust theoretical foundation for the clinical introduction of AB extract in knee osteoarthritis treatment [32].

**Figure 2.**
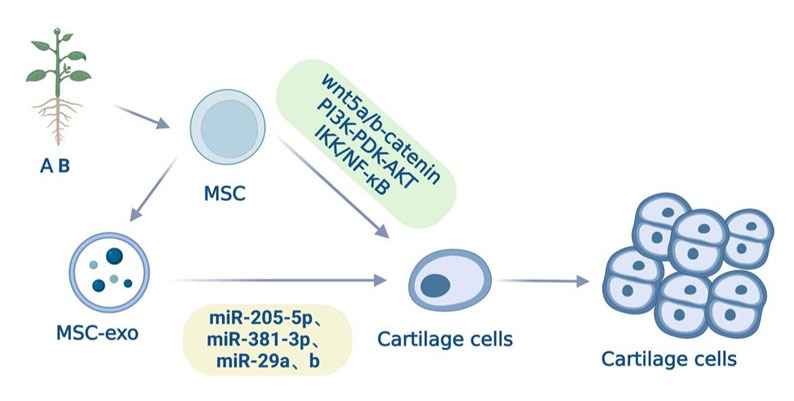
**AB can potentially act on MSCs in situ to promote the differentiation into chondrocytes and the secretion of MSC-exosomes to further achieve chondrocyte proliferation**. Exogenous stem cells can differentiate into cartilage and activate signaling pathways such as Wnt5a/b-catenin, PI3K-PDK-AKT, and IKK/NF-κB to improve chondrogenesis. In addition, the exosomes from these cells carry various miRNAs, such as miR-205-5p, miR-381-3p, miR-29a, and miR-29b, which can promote the process of the differentiation process of in situ stem cells into chondrocytes and contribute to chondrocyte proliferation. AB can improve the proliferation of chondrocytes in knee osteoarthritis patients and affect the cell cycle. AB can potentially act on in situ MSCs to improve the differentiation into chondrocytes and increase the secretion of MSC-exosomes to further enhance chondrocyte proliferation.

In light of the comprehensive review, it is evident that AB and MSCs share similarities in their ability to promote chondrogenesis. Consequently, AB holds the potential to influence MSCs in situ, thereby encouraging their differentiation into chondrocytes and the subsequent secretion of MSC-derived exosomes to foster chondrocyte proliferation (Fig. 2).

## 4. Improve subchondral bone microenvironment and its bone density to achieve anti-osteoporosis

Numerous studies have highlighted the close connection between knee osteoarthritis and the gradual deterioration of subchondral bone. To elaborate, the cartilage layer covers the surface of bones, and when osteoporosis develops in the distal femur and tibial plateau, increased pressure is exerted on the cartilage due to weight-bearing by the knee joint. Cartilage itself lacks the capacity to bear significant loads, making it susceptible to damage and inflammation when the knee joint is subjected to weight-induced stress. Hence, it becomes imperative to also address the issue of subchondral bone-related osteoporosis in the context of knee osteoarthritis treatment [33-34].

### 4.1 Anti-osteoporosis effects of MSCs

The pathogenesis of osteoporosis is inherently intricate, encompassing factors such as excessive bone resorption, insufficient bone formation, and impaired angiogenesis. Conventional treatment approaches often fall short in effectively addressing these complexities. In this context, MSCs emerge as pivotal players in bone formation. Research indicates that appropriately inducing osteogenic differentiation can lead to enhanced bone density [35]. Moreover, MSCs wield a paracrine effect, whereby they secrete numerous soluble factors linked to processes like angiogenesis, anti-apoptosis, anti-inflammation, and immunomodulation. This attribute enables MSCs to effectively counteract the progression of osteoporosis [36-37].

#### 4.1.1 Differentiation of MSCs into bone [38]

MSCs possess the capability to undergo osteogenic differentiation, contributing to bone formation. The underlying pathological mechanism of osteoporosis is closely tied to the diminished differentiation potential and overall quantity of MSCs present in human organs. In the treatment of osteoporosis, introducing MSCs through injections proves effective in replenishing MSC numbers and fostering bone development. Osteoporosis develops due to an imbalance between osteoclast and osteoblast quantities, which is correlated with osteogenic differentiation (OD). The Wnt/β-catenin signaling pathway plays a pivotal role in regulating OD. Notably, research has indicated that leucocyte cell-derived chemotaxin-2 (LECT2) expression is reduced in osteogenically differentiated MSCs. By downregulating LECT2, the Wnt/β-catenin pathway can be activated, thereby enhancing the capacity of MSCs to differentiate into bone cells [39]. In studies involving ovariectomized mice, MSCs administration has been found to increase trabecular density, trabecular volume, and the presence of trabeculae [40].

Furthermore, in cases where osteoporosis is induced by estrogen deficiency, tumor necrosis factor-alpha (TNF-α) has been shown to hinder MSC differentiation and bone formation. TNF-α primarily elevates the expression of p2y2 receptors through the c-Jun n-terminal kinase (JNK) and extracellular regulated protein kinase (ERK) pathways. Inhibiting p2y2 receptors results in the enhanced differentiation of MSCs into bone cells [41].

#### 4.1.2 MSC-exosomes can inhibit bone resorption and promote osteogenesis [42][43]

MSC-derived exosomes have been shown to possess the ability to suppress the secretion of IL-18 and IL-1β in osteoclasts treated with mercury (Hg), leading to the restoration of bone quantity in rats affected by streptomycin-induced Diabetic Osteoporosis. These exosomes effectively inhibit the activation of osteoclast NLRP3 inflammasomes, thereby reducing bone resorption and facilitating ossification [44]. Additionally, MSCs can promote osteogenesis by upregulating osteogenic markers, with RUNX2 being one of them. They can also inhibit adipogenic markers such as peroxisome proliferator-activated receptor γ (PPARγ) and osteoclast formation markers like nuclear factor, receptor activator of nuclear factor kappaB ligand (RANKL), consequently preventing adipogenesis or osteoclastogenesis. This modulation is achieved by activating specific pathways, namely sirtuin 1 (SIRT1) and the wingless associated MMTV integration site (Wnt). The role of MSC-AS1 involves elevating BMP2 expression through miRNA-140e5p, facilitating the differentiation of MSCs into osteoblasts, thus mitigating symptoms associated with osteoporosis [45].

Within MSCs, CDK8 plays a crucial role in controlling osteoclast formation and bone homeostasis. It contributes to bone resorption and homeostasis through the CDK8-STAT1-RANKL axis [46].

### 4.2 Anti-osteoporosis effects of AB

Inhibiting bone resorption and osteoclast development holds significant importance in the prevention and treatment of osteoporosis. In traditional Chinese medicine clinical practice, AB is widely utilized to address osteoporosis, serving as an anti-resorption agent for the condition [47]. AB has been shown to play multiple roles in bone health, including promoting cell differentiation into bone tissue, maintaining bone quantity, adjusting trabecular microstructure, and reducing serum biomarkers related to bone turnover.

To elaborate, AB polysaccharides exhibit the ability to hinder bone resorption and the emergence of osteoclasts by suppressing the RANKL signaling pathway. This action involves the downregulation of NFATc1 and c-Fos gene and protein expression induced by RANKL. Furthermore, AB suppresses NFATc1 transcriptional activity, and the phosphorylation of the MAPK pathway induced by RANKL signaling [48]. AB also demonstrates the potential to decrease the expression of key molecules such as CTSK, TRAc P, Integrin β3, and NFATc1 by inhibiting the MAPK pathway's phosphorylation. This inhibition of the MAPK pathway leads to a reduction in RANKL-induced osteoclast differentiation and function. Simultaneously, AB is observed to facilitate the differentiation of MSCs into bone tissue through the ERK-MAPK signaling pathway. Notably, AB induces MSC differentiation and osteogenic proliferation, resulting in an increase in the expression of BMP2, ALK, Runx2, and Osx mRNA in tested rats [49].

AB's osteoprotective effects have been evidenced in studies involving ovariectomized rats. These studies demonstrated that AB significantly induces osteoblast differentiation and enhances the expression of ALP activity, mineralization, and osteogenic markers in MC3T3-E1 cells. AB's interaction with the Wnt signaling pathway, particularly through the complex with BMP2, leads to enhanced expression of p-SMAD1, LEF-1, β-catenin, p-GSK-3β, and BMP2. This intricate interplay between BMP2/SMAD1 and Wnt/β-catenin signaling pathways contributes to the osteogenic effects observed [50]. Moreover, AB stimulates osteogenic proliferation in MC3T3-E1 cells, leading to increased ALP activity, mineral nodule formation, and expression of bone-related markers such as Bsp, Ocn, and Osx [51]. Experimental evidence also supports AB's protective effects on ovariectomized rats, demonstrating improvements in biomechanical properties, bone mineral content (BMC) of femurs, trabecular microstructure, and reduced serum markers of bone turnover after 13 weeks of treatment. This highlights AB's potential in mitigating bone loss associated with osteoporosis [52].

**Figure 3.**
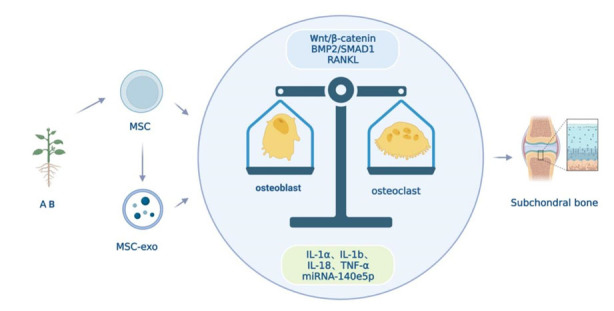
**Both MSCs and AB can balance osteoclasts and osteoblasts, promote bone formation, and inhibit bone resorption, thereby improving osteoporosis of subchondral bone**. AB can potentially achieve anti-osteoporosis by stimulating in situ stem cells. MSCs can differentiate into bone, and the injection of MSCs can effectively supplement the amount of MSCs and promote bone formation to treat osteoporosis. Osteoporosis is the result of an imbalance in the number of osteoblasts and osteoclasts, which is closely linked to osteogenic differentiation (OD). Stem cells can activate Wnt/β-catenin and BMP2/SMAD1 signaling pathways to regulate osteogenic differentiation and inhibit RANKL and other signaling pathways to suppress osteoclast formation and bone resorption. Moreover, miRNAs such as miRNA-140e5p in MSC-exosomes can upregulate BMP2 and promote MSCs’ osteogenic differentiation, thereby alleviating the development of osteoporosis. Stem cells can also inhibit the secretion of inflammatory factors such as IL-1a, IL-1b, IL-18, and TNF-α, to avoid bone loss. Both AB and MSCs have the effect of balancing osteoclasts and osteoblasts, promoting bone formation and inhibiting bone resorption, thus improving osteoporosis. AB can potentially achieve anti-osteoporosis by activating the above functions of in situ stem cells.

In summary, both MSCs and AB play pivotal roles in maintaining the balance between osteoclasts and osteoblasts, thereby promoting better bone development and inhibiting bone loss. AB's potential to stimulate in situ stem cells further emphasizes its capacity for anti-osteoporosis effects (Fig. 3).

## 5. Promote angiogenesis and improve microcirculation to achieve anti-thrombosis

Fascia and muscle tendons play a crucial role in human joints, serving not only to connect and stabilize joints while controlling their movement but also possessing essential metabolic functions. Within joints, there is a rich network of blood vessels that contribute to stabilizing microcirculation and producing synovial fluid. This synovial fluid serves to lubricate joints and provide chondrocytes with the necessary nutrition and metabolic support. Additionally, the density of subchondral bone in the joint is closely linked to microcirculation.

In the context of osteoarthritis, damage to the circulation around the joint is a significant contributing factor to cartilage erosion, chondrocyte cell death, and the progression of inflammation. As a result, it is crucial to address these vascular issues in arthritis treatment. This involves the restoration of damaged vascular endothelium, prevention of thrombosis, improvement of microcirculation within the soft tissues surrounding the joints and the subchondral bone, reinstatement of homeostasis in joint synovial fluid, and the maintenance of appropriate subchondral bone density.

In conclusion, the health of fascia, muscle tendons, and the vascular system surrounding joints is of utmost importance in the treatment of arthritis. Addressing the vascular aspects of joint health can have a profound impact on preventing further deterioration, promoting proper joint function, and alleviating the symptoms of arthritis.

### 5.1 Promotion of angiogenesis and inhibition of thrombosis by MSCs

MSCs have the potential for vascular epithelial differentiation, and MSC-exosomes can promote in situ vascular epithelial cell differentiation and repair vascular damage. MSCs can mediate BMP9 to induce angiogenesis by fostering the p53-Notch1 angiogenesis signaling axis via LncRNA-H19 [53]. Through the promotion of 17β-estradiol, MSCs express CD31, Akt, and p-Akt proteins, triggering signaling pathways such as MAPK and PI3K-Akt to achieve angiogenesis [54].

MiR-130a, a type of MSC-exosome, can increase endothelial cell proliferation, migration, and vessel formation. MiR-130a negatively regulates and targets phosphatase and tensin homologs, activating the PI3K/AKT signaling pathway. MSC-exosomes also accelerate neovascularization and bone regeneration in rat femur’s distal defects by delivering miR-146a, which promotes angiogenesis in endothelial cells by inhibiting Smad4 and nerve fiber-min2. MiR-21, also derived from MSC-exosomes, inhibits the neurogenic site Notch homologous protein 1/delta-like standard Notch ligand 4 pathway and enhances the expression of VEGFA and HIF1a. MSC-exosomes, such as MI-199b-3p and MI-125a-5p, significantly affect angiogenesis as well [55]. MSC-exosomes enhance angiogenesis in T2DM mice by inducing macrophage M2 phenotypic polarization through the JAK/STAT6 signaling pathway [56].

MSCs are capable of immunomodulation and microenvironmental improvement, as well as downregulating CRP, NETs, and pro-inflammatory cytokines, which helps reduce NETs-induced thrombosis formation [57].

### 5.2 AB’s improvement of microcirculation and prevention of thrombosis

**Figure 4.**
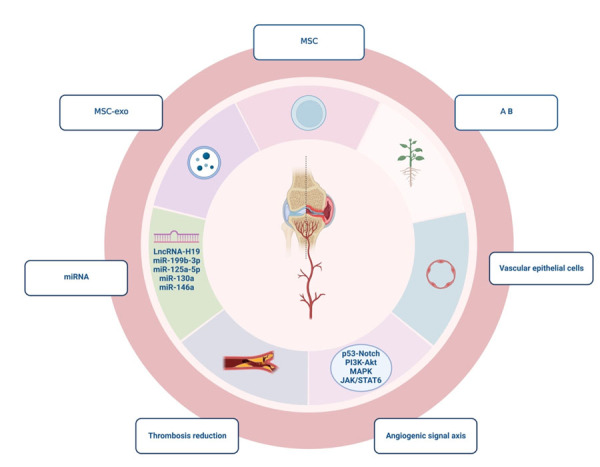
**Both AB and MSCs have the effect of promoting the proliferation of endothelial cells and preventing thrombosis, thereby improving the microcirculation around the knee joint**. AB can potentially achieve this by stimulating the *in situ* stem cells. Stem cells have the potential for vascular epithelial differentiation, and MSCs’ exosomes can promote in situ vascular epithelial cell differentiation and repair vascular damage. MSCs can increase endothelial cell proliferation, migration and vessel formation through LncRNA-H19, miR-130a, miR-146a, mi -199b-3p, and mi-125a-5p. MSC-exosomes can activate the signaling pathways such as p53-Notch, PI3K/AKT, MAPK, and JAK/STAT6, to achieve angiogenesis. MSCs are capable of immunomodulation, improving the microenvironment, and lowering the levels of CRP, pro-inflammatory cytokines and NETs, which helps reduce thrombosis formation caused by NETs. AB and MSCs both have the effect of promoting the proliferation of endothelial cells and preventing thrombosis, so as to improve the microcirculation around the knee joint in the treatment of osteoarthritis. AB can potentially achieve this by promoting the secretion of relevant exosomes through the activation of in situ stem cells.

AB, a traditional Chinese medicine, has been widely used to promote blood circulation and remove blood stasis. Among various formulations, the "Niu Xi Huo Xue Tang" formula is considered the safest and most effective remedy for preventing pulse thrombosis. AB not only modulates blood viscosity in both directions but also has the ability to promote the proliferation of vascular endothelial cells, as indicated by numerous studies [58]. AB pharmacological serum has been shown to enhance the proliferation of vascular endothelial cells, offering cellular protection, and effectively preventing LPS damage to these cells [59]. AB works by reducing downstream microthrombus formation through the inhibition of NF-κB, preventing endothelial cell oxidative damage and regulating tissue factor (TF) and plasminogen activator inhibitor-1 (PAI-1). ABPPk possesses the capability to inhibit the infiltration of polymorphonuclear leukocytes (PMNs) and the activation of matrix metalloproteinase-2/-9 (MMP-2/-9), thus effectively reducing the occurrence of thrombosis [60]. Both AB and MSCs have the potential to promote endothelial cell proliferation and prevent thrombosis, consequently improving the microcirculation around the knee joint. AB may achieve this effect by stimulating in situ stem cells (Figure 4).

## 6. Regulate estrogen

The reduction of estrogen levels in postmenopausal females becomes a significant risk factor for the development of osteoarthritis. The decline in estrogen can result in weakened skeletal muscle strength, joint instability, and heightened stress on articular cartilage and subchondral bone during movement, ultimately leading to cartilage degradation and joint degeneration, which culminate in osteoarthritis. Estrogen plays a crucial role in enhancing blood supply by dilating blood vessels in muscles, thereby increasing the delivery of nutrients to these tissues. Furthermore, estrogen has the capacity to significantly enhance muscle strength by improving the function of nerve cells. Hence, the regulation of estrogen holds a pivotal role in both preventing and rehabilitating knee joints [61].

### 6.1 MSCs’ regulation of estrogen

MSCs have demonstrated the potential to enhance ovarian function and stimulate estrogen secretion. MSC-exosomes containing miR-21 exhibit the capability to elevate estrogen levels in ovarian granule cells by facilitating YAP phosphorylation and LOXL2 through lats1 mediation [62]. Furthermore, MSCs can encourage the development of follicles, augment local ovarian vascularization, boost follicular and mesenchymal cell proliferation, and mitigate follicular atresia and apoptosis. Additionally, stem cells are able to enhance mitochondrial quality, promote mitochondrial DNA replication, decrease oxidative damage and cell apoptosis, and enhance the quality and overall quantity of mature oocytes that are capable of ovulation [63]. The presence of miR-644-5p within MSC-exosomes works to inhibit apoptosis in ovarian granule cells by targeting the cellular p53 protein, consequently restoring ovarian function [64]. In efforts to enhance angiogenesis, MSCs reduce the expression of inflammation-related genes IL8 and Tnf-α through EV20K and EV110K, while simultaneously increasing Igf1 and Vegf expression at the mRNA level, and αSMA and Vegf expression at the protein level. Additionally, MSCs hinder the apoptosis of follicular cells via the PI3K/AKT signaling pathway [65].

### 6.2 AB’s ovarian protection

AB is recognized in traditional Chinese medicine for its beneficial impact on kidney health. In the context of traditional Chinese medicine, the kidney's function encompasses female ovarian function as well. Research indicates that AB preparations have the ability to significantly enhance the elevation of serum diol (E2), with AB-contained cyasterone exhibiting estrogen-like effects [66]. Furthermore, AB plays a notable role in inhibiting follicular mitochondria-dependent apoptosis, which has implications for ovarian aging [67]. AB has been observed to increase the expression of Msx2 through the PKAIβ-mediated pathway, leading to enhanced MSC proliferation in ovariectomized rat tests [68]. Moreover, AB regulates the disorder of estrogen secretion through signaling pathways such as NF-κB, TNF, and AGE-RAGE. The use of shRNA targeting ln-cRNA SRA can inhibit the DHEA-induced production of inflammatory transmitters and the translocation of nuclear factor-κB in ovarian primary granule cells [69].

**Figure 5.**
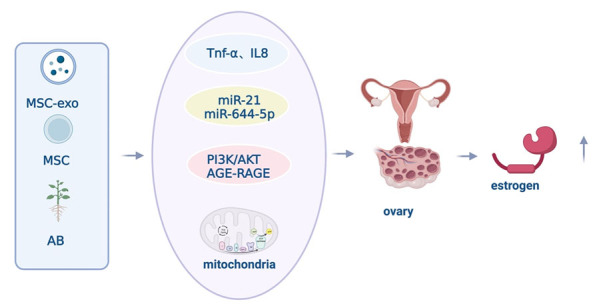
**Both MSCs and AB have the effect of improving ovarian function and promoting estrogen secretion**. AB can potentially affect in situ stem cells around the ovary to promote ovarian function and increase estrogen secretion. Stem cells can promote follicular development, increase local ovarian vascularization, enhance follicle and mesenchymal cell proliferation, reduce cell apoptosis and follicular atresia. They can also improve mitochondrial quality, increase mitochondrial DNA replication, reduce oxidative damage, and cell apoptosis, and increase the quantity and quality of ovulated mature oocytes. MiR-21 and miR-644-5p in MSC-exosomes can inhibit the apoptosis of ovarian granule cells and restore the ovarian function. MSCs also inhibit apoptosis of follicular cells through the PI3K/AKT and AGE-RAGE signaling pathways. AB and MSCs can improve ovarian function and promote estrogen secretion. AB can potentially activate the *in situ* stem cells around the ovary to promote ovarian function and avoid the reduction of serum estradiol (E2).

Both MSCs and AB demonstrate the potential to enhance ovarian function and promote estrogen secretion. AB holds the potential to influence in situ stem cells around the ovary, thereby promoting ovarian function and increasing estrogen secretion (Fig. 5).

## 7. Regulate intestinal flora

The human intestine harbors a diverse community of intestinal flora, numbering approximately 150 times the count of the human genome. This flora holds the capacity to influence the health status of the host by regulating various bodily functions, including the endocrine system, nervous system, and immune system, among others. It does so through mechanisms such as the gut-liver-bone axis and the gut-brain axis. The intestinal flora significantly impacts systemic inflammation and chronic conditions, including knee osteoarthritis. Manipulating the environment of the intestinal flora can lead to changes in the population of gram-negative bacteria, which are characterized by a high presence of LPS in their cell walls. This alteration can subsequently impact the LPS content in the body, triggering the production of inflammatory factors. This inflammatory response can hinder osteogenesis, encourage bone resorption, initiate cartilage degradation, give rise to osteoporosis, and ultimately contribute to the development of knee osteoarthritis. Consequently, the intestinal flora represents a potential therapeutic target for the treatment of knee osteoarthritis [70].

### 7.1 Oral administration of stem cell-loaded hydrogel microcapsules can restore intestinal flora and treat inflammation

The oral administration of stem cell-loaded hydrogel microcapsules (SC-HMs) offers advantages due to the lower stiffness of the central oil layer and the capsule as a whole. This characteristic enhances the retention and survival of MSCs even in the harsh gastric environment. Additionally, it promotes the controlled release of programmable cells during gastrointestinal peristalsis. In mouse models, SC-HMs have demonstrated the ability to aid in tissue recovery, diminish the infiltration of colonic macrophages (which secrete pro-inflammatory factors), and alleviate inflammatory bowel disease (IBD) [71]. MSCs released from SC-HMs can successfully colonize colonic crypts. Furthermore, metagenomic analyses have indicated that the use of SC-HMs can rectify imbalances in certain bacterial genera such as L. intestinalis, Lactobacillus (L.) gasseri, Bacteroides acidifaciens, and Lactobacillus reuteri. This optimization of microbial components and quantities has the potential to serve as an effective therapy for IBD [72].

### 7.2 Oral administration of AB supplements can reduce fecal E. coli counts

AB polysaccharide exhibits significant in vitro antibacterial activity and plays a role in preserving the balance of the intestinal microbiome in weaned piglets. To investigate this, AB total polysaccharide ARPS is extracted using a water extraction and alcohol precipitation method. Subsequent experiments measure the diameter of the in vitro antibacterial zone and the minimum antibacterial concentration against Escherichia coli, Salmonella, and Staphylococcus aureus. Furthermore, a feeding experiment is conducted with varying supplementary amounts of AB polysaccharide to assess its impact on the quantities of Lactobacillus, E. coli, and Bifidobacteria flora in piglets. Certain experimental findings indicate that AB polysaccharide indeed possesses a clear in vitro antibacterial activity and has the potential to maintain a balanced microbiome between the intestinal microbiota and weaned piglet bodies [73-74].

**Figure 6.**
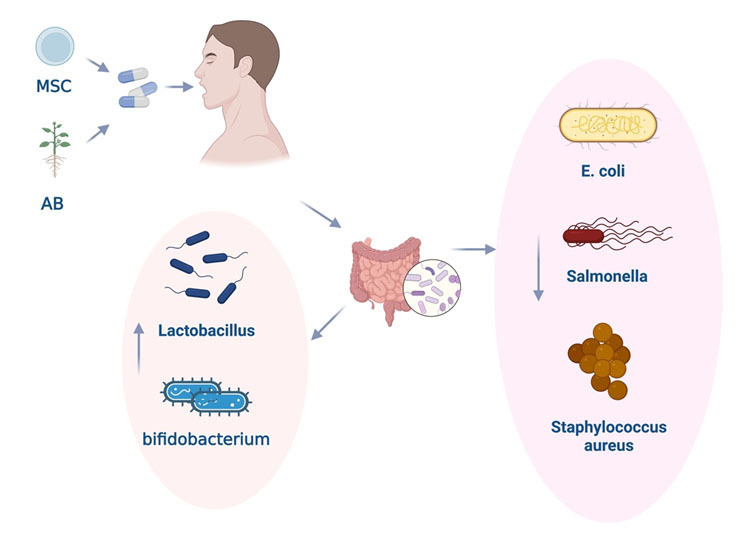
**Both MSCs and AB have the function of maintaining the balance of intestinal flora**. AB can possibly activate the MSCs in the intestine to regulate the intestinal flora. Oral administration of stem cell-loaded hydrogel microcapsules can improve the disorders of specific bacterial genera, including *Bacteroides acidifaciens, Lactobacillus (L.) gasseri, Lactobacillus reuteri,* and *L. intestinalis*. This improvement optimizes microbial composition and abundance. Oral administration of AB supplements can reduce Escherichia coli, Salmonella, and Staphylococcus aureus in feces; the increasing number of lactobacillus and bifidobacteria can maintain the microbiome balance between the intestinal microbiota and the body. Both MSCs and AB have the function of maintaining the balance of intestinal flora. AB can potentially activate the MSCs in the intestine and promote the secretion of MSC-exosomes to affect the intestinal flora.

Both MSCs and AB contribute to the equilibrium of intestinal flora. Exosome-based drugs can target bones to counteract osteoblast formation or bone resorption in inflammatory bowel diseases [75]. It's plausible that AB may activate MSCs in the intestine to regulate the intestinal flora (Fig. 6).

## 8. Conclusion

During early human embryonic development, a blastocyst forms first, followed by the development of the heart after 3-4 weeks, and then nerves after 5 weeks. Tissues such as organs, bones, and muscles develop subsequently. This progression highlights the importance of the blastocyst-like structure, which serves as a foundational structure for the entire body and organs. Stem cells are commonly found within this structure, playing a crucial role in maintaining organ metabolism and renewal.

In addition to stem cell transplantation, activating endogenous cells is crucial for combating age-related diseases. Activating the mesenchymal tissue system is a fundamental step for regeneration during the aging process. In traditional Chinese medicine, the "Sanjiao" (triple energizer) refers to the largest organ derived from the MSC system during embryonic development. It produces functional cells crucial for embryonic development. As the body ages, the triple energizer mesenchymal stem cell system becomes less dense and more obstructed, leading to mechanical instability and disease formation. The knee joint, as a functional organ within this system, relies on it for nutrition, circulation, and metabolism. Damage to the fascia and capsule structure or issues like thrombosis and contracture can lead to knee joint and cartilage lesions.

**Figure 7.**
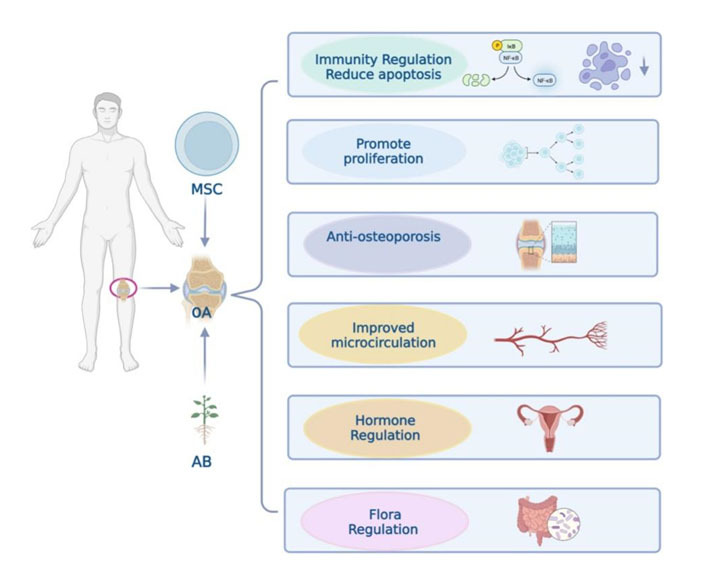
**MSCs and AB have efficacy in treating knee osteoarthritis in six aspects**. There are six similar functions of MSCs and AB in the treatment of osteoarthritis: 1. reduce the cartilage apoptosis through immunomodulation; 2. promote chondrocyte proliferation; 3. improve subchondral bone microenvironment to achieve anti-osteoporosis; 4. promote angiogenesis and improve microcirculation to achieve anti-thrombosis; 5. promote the follicular cells activity to increase the estrogen secretion; 6. optimize intestinal flora. Because of the similar mechanism between MSCs and AB in the treatment of knee osteoarthritis, AB possibly achieves the above effects by activating the body’s *in situ* cells. The joint application of AB and stem cells can potentially produce a greater therapeutic effect on knee osteoarthritis.

Expanding the research focus from cartilage and subchondral bone to the triple energizer mesenchymal stem cell system around the knee joint is essential. This system, encompassing blood vessels, nerves, lymph, and body fluids, acts as a circulating medium that provides circulation, metabolism, and nutrition to the body's functional organs. Traditional medicine such as AB has shown efficacy in treating liver and kidney diseases. MSCs are essential for human growth and development, differentiating into various tissues. The triple energizer mesenchymal stem cell system covers interstitial tissues like fascia, body fluids, fat, and capsule structures, playing a role in providing nutrients and regulating tissue metabolism.

To treat knee osteoarthritis effectively, it's important to consider the knee joint as a holistic entity. The triple energizer mesenchymal stem cell system around the knee joint can be rehabilitated against relaxation, injury, and obstruction using methods like drugs, massage, acupuncture, small acupuncture scalpel, fomentation, and functional training. This holistic approach is a practical and fundamental way to address knee osteoarthritis.

The knee joint operates as a self-sustaining homeostatic system, consisting of various components like bones, cartilage, tendons, fascia, synovium, synovial fluid, bursa, and fat pad. Both AB and MSCs can target any of these components due to their origins and pharmacological effects. Given that AB can potentially influence in situ stem cells, it's reasonable to assume that AB-induced MSCs are also effective in treating osteoarthritis. Nonetheless, further research is needed to confirm this hypothesis (Fig. 7).

MSCs and AB share some similarities in their mechanisms, particularly in promoting tissue regeneration and modulating various cellular processes. However, there is currently a lack of proper studies that directly compare the effects of MSCs and AB in the treatment of arthritis. Therefore, conducting a comprehensive analysis and comparison of their mechanisms is both necessary and urgent to understand their potential synergy or differences.

Safety is of paramount importance in any form of treatment. In the case of MSCs, their safety profile has been clinically established over the past 19 years, with extensive use in various conditions. Clinical data analysis of arthritis patients who received MSC treatment has shown significant symptom reduction without serious adverse events. Similarly, AB, as a traditional Chinese medicine with a history of nearly 2000 years, has been used safely in various contexts. However, new methods of administration and usage of AB may require additional safety verification.

Current administration methods for MSCs in arthritis treatment include intravenous infusion, injections around joint attachment points, and gel capsule drug delivery. For AB, administration methods include oral consumption, topical application, and injection of purified components around joint acupuncture points. The specific dosage, timing, and administration methods need to be tailored to the individual patient's condition, extent of joint damage, and clinical requirements.

The unique pluripotent differentiation capabilities of MSCs make them invaluable in regenerative medicine. However, exploring how to enhance their effectiveness in treating target diseases, leveraging the synergistic effects of traditional Chinese medicine on stem cell differentiation, establishing standardized preparation systems for traditional Chinese medicine-induced stem cells, and developing safe and effective clinical evaluation frameworks are all important research directions that can contribute to the effective treatment of diseases by combining stem cells and traditional Chinese medicine.

In conclusion, the convergence of stem cells and traditional Chinese medicine holds promise for novel and effective therapeutic approaches. Comprehensive studies and well-designed trials are needed to fully understand the potential benefits, interactions, and safety of combining these approaches in treating various diseases, including arthritis.
